# Supplementary material for: COZOID: contact zone identifier for visual analysis of protein-protein interactions
Source: BMC Bioinformatics. 2018 Apr 6;19:125. doi: 10.1186/s12859-018-2113-6 (PMC5889581; doi:10.1186/s12859-018-2113-6)
Supplement: Supplementary file 2 — Software build. Executable binary file of the software tool. (ZIP 118,784 kb) [file 12859_2018_2113_MOESM2_ESM.zip › cozoid/java/docs/beansbinding-1.2.1-doc.zip!/org/jdesktop/swingbinding/adapters/BeanAdapterBase.html]

BeanAdapterBase (Beans Binding)


---


|  |  |  |
| --- | --- | --- |
| **Overview** \| **Package** \| **Class** \| **Use** \| **Tree** \| **Deprecated** \| **Index** \| **Help** | |  |
| **PREV CLASS**   **NEXT CLASS** | **FRAMES**    **NO FRAMES**     **All Classes** |  |
| SUMMARY: NESTED \| FIELD \| CONSTR \| METHOD | DETAIL: FIELD \| CONSTR \| METHOD |  |


---


## org.jdesktop.swingbinding.adapters Class BeanAdapterBase

```
java.lang.Object
  org.jdesktop.swingbinding.adapters.BeanAdapterBase
```

**Direct Known Subclasses:**: AbstractButtonAdapterProvider.Adapter, JComboBoxAdapterProvider.Adapter, JListAdapterProvider.Adapter, JSliderAdapterProvider.Adapter, JSpinnerAdapterProvider.Adapter, JTableAdapterProvider.Adapter, JTextComponentAdapterProvider.Adapter

---

``` public class BeanAdapterBase extends java.lang.Object ```

---

| **Field Summary** | |
| --- | --- |
| `protected  java.lang.String` | `property` |


| **Constructor Summary** | |
| --- | --- |
| `protected` | `BeanAdapterBase(java.lang.String property)` |


| **Method Summary** | |
| --- | --- |
| `void` | `addPropertyChangeListener(java.beans.PropertyChangeListener listener)` |
| `void` | `addPropertyChangeListener(java.lang.String property, java.beans.PropertyChangeListener listener)` |
| `protected  void` | `firePropertyChange(java.lang.Object oldValue, java.lang.Object newValue)` |
| `java.beans.PropertyChangeListener[]` | `getPropertyChangeListeners()` |
| `java.beans.PropertyChangeListener[]` | `getPropertyChangeListeners(java.lang.String property)` |
| `protected  boolean` | `isListening()` |
| `protected  void` | `listeningStarted()` |
| `protected  void` | `listeningStopped()` |
| `void` | `removePropertyChangeListener(java.beans.PropertyChangeListener listener)` |
| `void` | `removePropertyChangeListener(java.lang.String property, java.beans.PropertyChangeListener listener)` |

**Methods inherited from class java.lang.Object**

`clone, equals, finalize, getClass, hashCode, notify, notifyAll, toString, wait, wait, wait`

**Field Detail**

### property

```
protected final java.lang.String property
```


**Constructor Detail**

### BeanAdapterBase

```
protected BeanAdapterBase(java.lang.String property)
```


**Method Detail**

### listeningStarted

```
protected void listeningStarted()
```

---


### listeningStopped

```
protected void listeningStopped()
```

---


### isListening

```
protected final boolean isListening()
```

---


### addPropertyChangeListener

```
public final void addPropertyChangeListener(java.beans.PropertyChangeListener listener)
```

---


### removePropertyChangeListener

```
public final void removePropertyChangeListener(java.beans.PropertyChangeListener listener)
```

---


### getPropertyChangeListeners

```
public final java.beans.PropertyChangeListener[] getPropertyChangeListeners()
```

---


### addPropertyChangeListener

```
public final void addPropertyChangeListener(java.lang.String property,
                                            java.beans.PropertyChangeListener listener)
```

---


### removePropertyChangeListener

```
public final void removePropertyChangeListener(java.lang.String property,
                                               java.beans.PropertyChangeListener listener)
```

---


### getPropertyChangeListeners

```
public final java.beans.PropertyChangeListener[] getPropertyChangeListeners(java.lang.String property)
```

---


### firePropertyChange

```
protected final void firePropertyChange(java.lang.Object oldValue,
                                        java.lang.Object newValue)
```


---


|  |  |  |
| --- | --- | --- |
| **Overview** \| **Package** \| **Class** \| **Use** \| **Tree** \| **Deprecated** \| **Index** \| **Help** | |  |
| **PREV CLASS**   **NEXT CLASS** | **FRAMES**    **NO FRAMES**     **All Classes** |  |
| SUMMARY: NESTED \| FIELD \| CONSTR \| METHOD | DETAIL: FIELD \| CONSTR \| METHOD |  |


---
